# Subjective Experiences of Pregnancy, Delivery, and Nursing in Transgender Men and Non-Binary Individuals: A Qualitative Analysis of Gender and Mental Health Concerns

**DOI:** 10.1007/s10508-023-02787-0

**Published:** 2024-01-16

**Authors:** Felicitas A. O. K. Falck, Cecilia M. U. Dhejne, Louise M. M. Frisén, Gabriela M. Armuand

**Affiliations:** 1 Centre for Psychiatry Research, Department of Clinical Neuroscience, Karolinska Institutet, Stockholm, Sweden; 2 ANOVA, Karolinska University Hospital, 171 76 Stockholm, Sweden; 3 Psychiatry Southwest, Karolinska University Hospital in Huddinge, Stockholm, Sweden; 4 Department of Medicine, Huddinge, Karolinska Institutet, Stockholm, Sweden; 5 School of Health and Welfare, Faculty of Health Sciences 1, Dalarna University, Falun, Sweden

**Keywords:** Gender dysphoria, Gender congruence, Gender identity, Post-partum depression, Coming-out process, Breast feeding

## Abstract

Studies of how gender-diverse individuals experience pregnancy, childbirth, and nursing remain few, mainly focus on the US and contain scarce information about mental health concerns peri-partum. This hinders informed reproductive health decisions and counseling. We used in-depth interviews to examine how gestational gender-diverse individuals in Sweden experience the process of planning and undergoing pregnancy, delivery, and nursing. In total, 12 participants, identifying on the masculine side of the gender spectrum or as non-binary, who had attended Swedish antenatal care and delivered a live birth, were included in the study. Data were analyzed using qualitative thematic content analysis. The analysis resulted in one overarching theme: sustaining gender congruence during pregnancy and three main categories: (1) considering pregnancy; (2) undergoing pregnancy and childbirth; and (3) postnatal reflections. The association between childbearing and being regarded as female permeated narratives. Participants renegotiated the feminine connotations of pregnancy, accessed gender-affirming treatment, and concealed their pregnancy to safeguard their gender congruence. Mis-gendering and breast enlargement triggered gender dysphoria. Social judgment, loneliness, information shortages, hormonal influence and cessation of testosterone increased gender dysphoria and strained their mental health. Depression exacerbated gender dysphoria and made it harder to claim one’s gender identity. Dissociation was used to handle a feminized body, vaginal delivery, and nursing. Pregnancy was easier to envision and handle after masculinizing gender-affirming treatments. The results deepen the understanding of gender dysphoria and may be used to inform reproductive counseling and healthcare development. Research outcomes on mental health concerns provide a basis for further research.

## Introduction

Although many gender-diverse individuals want to become parents (Stolk et al., 2023), their reproductive needs are often overlooked (Brandt et al., 2019; Falck et al., 2021). Gender-diverse individuals (e.g., individuals who do not identify with the gender that was assigned to them at birth), may seek gender-affirming treatment to align their body with their gender identity (Coleman et al., 2022). The majority of those who were assigned female at birth retain their uterus (Cipres et al., 2017; Kailas et al., 2017). While these individuals have the reproductive anatomy and physiology for gestational pregnancy (Obedin-Maliver & Makadon, 2016), emotional aspects, gender norms, medical factors as well as legal restrictions may provide obstacles to bearing a child (Cheng et al., 2019). Still, gender-diverse individuals assigned female at birth undergo intended and unintended pregnancy, including after gender-affirming hormonal treatment and chest surgery (Chen et al., 2018; Ellis et al., 2015; Falck et al., 2021; Hoffkling et al., 2017; van Amesfoort et al., 2023; Wierckx et al., 2012).

Data on pregnancy rates and birth rates in female-assigned gender-diverse individuals are scant, but pregnancy may be of relevance for a substantial number. Two studies reported that, respectively, 17% and 12% of the gender-diverse individuals assigned female at birth had been pregnant at least once (Light et al., 2018; Moseson et al., 2021). Moseson et al. calculated a birth rate of 39% from 433 pregnancies, while Light et al. (2018) reported a birth rate of 29% in 60 pregnancies. Leonard et al. (2022) found that 498 individuals who had given birth in California between 2016 and 2019 were registered as fathers on the birth certificate of their children and reported no difference in obstetrical or birth outcomes compared to cisgender mothers with a male partner. Moreso, gender-diverse gestational individuals report lactation after giving birth and 33% exclusively nurse their child (Yang et al., 2023), although the choice to nurse may be compromised by gender dysphoria, chest masculinization surgery and a lack of support (MacDonald et al., 2016; Wolfe-Roubatis & Spatz, 2015; Yang et al., 2023).

The proportion of individuals in the general population who identify as transgender is estimated to be 0.3–0.5% (Coleman et al., 2022). In a population-based study in Stockholm, Sweden, 0.4% of those who were assigned female at birth stated that they would like to receive hormones or surgery to align their body with their gender (Åhs et al., 2018). While the proportion of assigned females who seek gender-affirming health care or are diagnosed with gender dysphoria range from 0.0007% to 0.019% (Goodman et al., 2019), an increase in the number who seek assessment for gender dysphoria is reported from many countries (Goodman et al., 2019; Indremo et al., 2021) and is especially pronounced among those assigned female at birth (Goodman et al., 2019) (e.g., among individuals who have the capacity to undergo pregnancy). Still, clinicians lack knowledge on how gender-diverse individuals with an intention of pregnancy should be evaluated for gender-affirming care (Falck et al., 2021) and how to support them during pregnancy and child birth (Falck et al., 2021; Johansson et al., 2020; Qin et al., 2021). While this lack of knowledge partly stems from insufficient training (Obedin-Maliver, 2015), it can also be attributed to limited research on pregnancy-related needs among transgender individuals (McCracken et al., 2022) including whether pregnancy can be reconciled with a masculine gender identity or not.

The concept gender diverse encompasses transgender men and transmasculine individuals who were assigned female at birth based on their reproductive anatomy but identify on the masculine side of the gender spectrum, as well as non-binary individuals who position their gender identity beyond or between what is normatively perceived as feminine and masculine (Riggs & Bartholomaeus, 2018; Tasker & Gato, 2020). Gender-diverse individuals can be described as gender incongruent as their gender identity contradicts their birth sex phenotype and/or how others perceive their sex/gender. When this incongruence causes persistent distress that impairs daily functioning, it is labeled gender dysphoria (Davy & Toze, 2018). Some gender-diverse individuals seek gender-affirming treatment to reduce gender dysphoria. Gender-affirming treatment may involve any combination of testosterone treatment, surgical removal of breast tissue to create a masculine silhouette, or genital surgery, which usually consists of an oophorectomy, hysterectomy, vaginectomy and the construction of a penis (Coleman et al., 2022). Female-assigned gender-diverse individuals report improvement in body satisfaction and depressive symptoms after testosterone treatment (Fisher et al., 2016) and have a lower odds of anxiety and depressive symptoms compared to those without treatment (Owen-Smith et al., 2018; St. Armand et al., 2011) and a better body image (van de Grift et al., 2017). Likewise masculinizing chest surgery and genital surgery have been associated with lower odds of body dissatisfaction, symptoms of depression and anxiety (Bustos et al., 2021; Owen-Smith et al., 2018). While expert opinion holds that gender-affirming treatment among transgender men and women decreases gender dysphoria (Coleman et al., 2022; Murad et al., 2010), improves psychological well-being (Nguyen et al., 2018; van Leerdam et al., 2023) and quality of life (van Leerdam et al., 2023), such treatments may limit reproductive options.

Testosterone suppresses ovarian activity as indicated by attenuated estradiol levels that remain stable in the early follicular range at 46 pg/mL after three months (Sommer et al., 2008), 36 pg/mL after four months (Slabbekoorn et al., 1999) and 28 pg/mL after 60 months (Carrillo et al., 2010) of testosterone treatment. Although testosterone inhibits ovulation among those who have ovaries (Meyer et al., 1986; Taub et al., 2020), it has been shown to resume if testosterone administration is stopped (T'Sjoen et al., 2019). Ovaries exposed to testosterone have been found to exhibit histological changes similar to polycystic ovarian syndrome after 6 months (Grynberg et al., 2010) as well as 21 months of treatment (Pache et al., 1991). However, other studies have not found this association after 16 weeks (Caanen et al., 2015) or 58 weeks of testosterone treatment (de Roo et al., 2017).

Similarly, studies on the outcomes of assisted reproduction in gender-diverse individuals show conflicting results. The number of harvested oocytes was larger among transgender men who had used testosterone for 44 months on average as compared to a cisgender control group in one study (Leung et al., 2019). Other studies did not indicate any difference in the number of antral follicles among transgender men exposed to testosterone for 2–9 years (Adeleye et al., 2019) or 16 weeks (Caanen et al., 2015) as compared to testosterone-naïve subjects. While the long-term effects of testosterone on ovarian function and fertility are yet to be determined, gender-affirming genital surgery makes the individual unable to carry a pregnancy (Cheng et al., 2019). The fact that such gender-affirming genital surgery or sterilization, involving the destruction or removal of the fallopian tubes, is still required to change legal gender in seven countries in Europe and Central Asia (Transgender Europe, 2022), illustrates that laws and regulations still impact reproductive health options (Dunne, 2017; Stuyver et al., 2020).

In recent years, the reproductive rights of gender-diverse individuals have been increasingly recognized (Gonzalez-Salzberg, 2018; World Health Organization, 2014) and the World Professional Association for Transgender Health recommends that counseling about reproductive options should take place before gender-affirming treatment is initiated (Coleman et al., 2022). Ideally such counseling should build on research of how gender-diverse individuals experience pregnancy. Although growing in numbers, few studies have had this focus. Existing qualitative data on the association between pregnancy and femininity among transgender individuals note that stigma and discrimination lead to limited disclosure of pregnancy intentions and loneliness (Besse et al., 2020; Charter et al., 2018; Ellis et al., 2015). While pregnancy could be reconciled with a masculine gender identity of some transgender men, others experienced increased gender dysphoria (Brandt et al., 2019; Charlton et al., 2021; Charter et al., 2018; Ellis et al., 2015; Light et al., 2014), exacerbated by discontinued testosterone use (Charter et al., 2018; Ellis et al., 2015), breast enlargement (Charter et al., 2018; Ellis et al., 2015; Falck et al., 2021; MacDonald et al., 2016) and judgmental treatment (Charter et al., 2018; Ellis et al., 2015; Falck et al., 2021; MacDonald et al., 2016).

Existing studies on experiences of pregnancy and childbirth among gender-diverse individuals have mainly been conducted in North America (Besse et al., 2020; Chen et al., 2018; Ellis et al., 2015; Hoffkling et al., 2017; Light et al., 2014), with only a very limited number of studies from Europe (Falck et al., 2021; van Amesfoort et al., 2023) and Australia (Charter et al., 2018). As assessment procedures for gender dysphoria (Coleman et al., 2022), patient costs for gender-affirming care (Dowshen et al., 2019), as well as laws (Falck & Bränström, 2023; Transgender Europe, 2022), and attitudes (Falck & Bränström, 2023) toward transgender individuals vary between countries and are likely to affect experiences of pregnancy and childbirth, research from different contexts is needed (Falck et al., 2021). Another important gap in the current literature is the shortage of research on the mental health of gender-diverse individuals during the peri- and post-partum period (Kirubarajan et al., 2022). Transgender individuals have a larger burden of mental health problems as compared to the general population (Bockting et al., 2013; Socialstyrelsen, 2020; Veale et al., 2017), are likely to have a greater risk of post-partum mental health problems (Kirubarajan et al., 2022) and experience barriers to mental health care during post-partum depression (Hoffkling et al., 2017), indicating the need to understand what factors that increase the risk of mental health problems in association with pregnancy and childbirth. From a patient perspective, evidence-based information of how gender-diverse individuals experience pregnancy, childbirth, and nursing, can help individuals with gender dysphoria make informed decisions on whether they want to pursue pregnancy or not, save oocytes, and when to initiate gender-affirming treatments. It may also deepen the understanding of gender dysphoria among clinicians, strengthen their assessment for and delivery of gender-affirming care, reproductive counseling, and support during pregnancy and post-partum. Consequently, the objective of this study was to examine how transgender men and non-binary individuals in Sweden experience pregnancy, delivery and nursing.

### Gender-Affirming Care and Reproductive Regulations in Sweden

In Sweden, individuals who seek gender-affirming treatment are assessed for a gender dysphoria diagnosis according to ICD-10 (World Health Organization, 2016). Evaluations take place at specialized gender clinics, involving a psychiatrist and a psychologist. Gender-affirming testosterone treatment and chest masculinization surgery require a gender dysphoria diagnosis and are funded by general health coverage (Dhejne et al., 2014). Access to such treatment is not based on the informed consent model, where the patient makes the decision to initiate treatment (Coleman et al., 2022), but is subject to the decision of the gender team (Linander et al., 2019). Individuals who identify according to the gender binary as men or women, but not as non-binary, may obtain legal gender recognition and gender-affirming genital surgery upon application to the National Board of Health and Welfare (Dhejne et al., 2014). Until 2013, legal gender recognition was only allowed if the individual was sterile (Dhejne et al., 2014). Since the law was amended, fertility preservation is offered before the initiation of gender-affirming treatment and in vitro fertilization is available (Armuand et al., 2017). However, adoption is rare and surrogacy is not allowed (Gunnarsson Payne & Handelsman-Nielsen, 2023), which is a contrast to the US where surrogacy is available (Smietana et al., 2021). Egg donation to a spouse was not permitted before 2019 in Sweden. Consequently, until recently and when the interviews for this study were performed, female-assigned gender-diverse individuals could only have a child from their own germ cells by undergoing pregnancy themselves.

As pregnancy is not always planned, it is also worth noting that abortions in Sweden are readily available as they are covered by national health insurance, unrestricted until the end of gestational week 18 and often performed by a midwife (Endler et al., 2020).

## Method

### Participants

Participants were recruited through purposive sampling and were eligible to participate in the study if they (1) identified on the masculine side of the gender spectrum or as non-binary before pregnancy, (2) had been referred for a diagnostic evaluation of gender dysphoria before or during pregnancy, and (3) had given birth after the sterilization requirement to change legal gender was removed from national law in 2013. The community organizations RFSL and RFSL Youth, which advocate for the health and rights of lesbian, gay, bisexual, and gender-diverse individuals, helped recruit participants to the study. The primary author visited the annual meeting of RFSL to reach volunteers and staff working in rural as well as urban areas with information about the study. RFSL and RFSL Youth then provided written information about the study and its purpose in social media and on their webpages to reach potential participants. Professionals in the Swedish Gender Teams recruited participants at their clinics by posting written information about the study, its purpose and contact details to the researchers in waiting areas. In addition, former study participants informed their peers about the study by word of mouth. Potential participants were then contacted over email by the primary author to provide them with written information about the study and its purpose. This information was also provided in written form as well as orally before the face-to-face interviews. The purpose of the study was presented to participants. It was to explore how transgender men and non-binary individuals experience pregnancy, delivery, and nursing as well as related healthcare encounters to deepen the understanding of gender dysphoria and help improve the quality of care. Potential participants were also informed about what procedures that were in place to ensure confidentiality. After they had been able to ask questions, they confirmed their consent to participate in writing.

### Procedure and Measures

Twelve in-depth face-to-face interviews were carried out from December 2016 to January 2019. Interviews lasted 55–112 min (mean, 83 min), were digitally recorded, and transcribed verbatim. They were conducted two months to two years after the last childbirth of each of the 12 participants. The location of the interviews was chosen by the participants, and the primary author traveled across Sweden to conduct them, reaching urban and rural areas. Demographic information on age, occupation, relationship status at conception, gender identity, gender dysphoria diagnosis and gender-affirming treatments was collected at the outset of each interview. All interviews then started with the question: “What was it like for you to undergo pregnancy as a man/transmasculine/non-binary individual?.” Based on an interview guide, internal and external experiences during the preconception phase, pregnancy and childbirth, as well as baby-feeding practices were explored.

Interviews were unstructured and retrospective. The unstructured interview format was chosen since it gives participants control over how much information they convey, to account for the potentially sensitive topic of the interviews, and since it allows for detailed accounts (Riessman, 2008), increasing the depth of the data (Corbin & Morse, 2003). The interviews were retrospective as we wanted to capture the whole process of undergoing pregnancy, from the initial thoughts of considering it, to the time after delivery. This also allowed us to find enough participants for the study. The interviewer followed the narrative of each respondent, asking for clarifications when needed. Field notes were taken. Saturation was reached at nine interviews, after which three additional ones were conducted. Interactions with healthcare providers have been presented in a separate article (Falck et al., 2021) and are therefore not the focus of this text.

### Data Analysis

Since the area of research was understudied, an inductive approach was chosen (Graneheim et al., 2017). The data was analyzed using thematic content analysis (Burnard, 1991; Burnard et al., 2008), which enabled us to identify, organize, analyze and present the themes of the interviews in a detailed manner, using NVivo software. Qualitative research recommendations (Tong et al., 2007) and established criteria for the analysis and presentation of unstructured interviews (Lincoln & Guba, 1985) were used. After transcription, all texts were read three times for a preliminary overview. Phrases relevant for the study aim were then identified, coded, and categorized based on similar content, creating main and sub-categories. The transcripts were reread 4–6 additional times, going back and forth between the interview transcripts and the categories to avoid misinterpretations and loss of information, deepen, merge, and regroup the categories, and develop and overarching theme based on the data. The first author performed all interviews, the initial coding of the data, and took the lead in the analysis. Once preliminary codes and categories had been formulated, the final author took part in the analysis to help refine and regroup these. The second and third authors were then invited to discuss the data to reach final consensus on main and subcategories as well as the overarching theme. There were no major differences of interpretation between the authors, supporting the reliability of the analysis. Interviews focused on pregnancies carried to term after July 2013, however data from previous and ongoing pregnancies were included when relevant. Quotations were chosen to illustrate the results, as presented in the results section. Square brackets have been used to indicate where clarifications were necessary and text excisions have been marked using three dots.

The research team included researchers with clinical expertise in transgender health care, psychiatry, midwifery, and knowledge of gender theory and human rights. Since the positionality of researchers affects research questions and results (Malterud, 2001), the study was designed in consultation with the organizations RFSL and RFSL Youth.

## Results

### Demographic Characteristics

The age of the 12 participants was 21–40 years, with a mean age of 31. Two participants originated from outside of Sweden. Half of the participants lived in a major city and half in a small town or rural area. Employment was high as eleven participants were working, studying, or on parental leave. All had been referred for an evaluation of gender dysphoria before giving birth, as this was an inclusion criterium, and all but one had initiated their diagnostic evaluation before childbirth. Five participants had undergone both testosterone treatment and chest surgery, three had taken testosterone only, while one had undergone chest surgery only. Three participants had not had any gender-affirming treatments but were aiming to obtain it. Two of those who had not initiated any gender-affirming treatment were still in assessment for such treatment by the gender team. The duration of testosterone treatment varied from six months to seven years among the eight participants who had taken testosterone prior to their pregnancy. No participant regretted their gender-affirming treatments. None had undergone gender-affirming genital surgery.

No participant had planned their last pregnancy carried to term with a partner who identified as female. Two participants had planned their last pregnancy carried to term with partners who were assigned female at birth but identified on the masculine side of the gender spectrum or as non-binary. Three participants had planned their last pregnancy carried to term as single. Five participants had planned their last pregnancy carried to term with a cis-gender male partner. One of these participants had separated since the birth of his last child and was currently undergoing an unplanned pregnancy. While the interview with this individual focused on his last pregnancy carried to term, his current experiences of undergoing an unplanned pregnancy were also included in this manuscript. Two participants had given birth to an unplanned child. Among those who had an unplanned pregnancy, one individual conceived through non-consensual sex, while two conceived through consensual intercourse.

During the peri-partum period of their last bourn child, eight participants had at least one partner who was also a parent to their child, while two participants went through the peri-partum period as single and without a co-parent. In addition one participant was single but shared the parental responsibilities with a former partner, while another participant was in a relationship with a new partner who was not sharing the parental responsibilities with the participant. Four participants had given birth to more than one child. Eight participants had given birth to one child, and one of these was currently pregnant and about to give birth to his second child.

Participants used varying combinations of concepts to describe their gender. Eight participants regarded themselves as men, while four saw themselves as non-binary, leaning toward masculinity. For reasons of consistency and anonymity, participants are all referred to as gender diverse in this manuscript; however, preferred pronouns and fictive names are used. Demographic information is presented in Tables 1 and 2.

**Table 1:** Sociodemographic data on age, gender identity, evaluation and gender-affirming treatment, employment status, and urbanicity (n = 12)

Variable	*n* (%)
*Age at time of study*	
Mean (in years)	31
Range (in years)	21–40
	*n* (%)
*Gender identity*	
Man and/or transman	8 (66.7)
Transmasculine and non-binary	3 (25)
Non-binary	1 (8.3)
*Completion of diagnostic evaluation at time of interview*	
Yes	10 (83.3)
No	2 (16.7)
*Gender-affirming treatment prior to pregnancy*	
Testosterone* and chest surgery	5 (41.7)
Testosterone* only	3 (25.0)
Chest surgery only	1 (8.3)
No treatment	3 (25.0)
Regrets of gender-affirming treatments	0 (0.0)
*Employment status*	
Employed	11 (91.7)
Unemployed	1 (8.3)
*Place of living at time of childbirth*	
Major city	6 (50.0)
Town/rural area	6 (50.0)

* Duration of testosterone treatment between 6 months and 7 years

**Table 2:** Data on conception, relationship status, delivery, and children carried to term (n = 12)

Variable	*n* (%)
*Way of conception*	
Intercourse	6 (50.0)
Insemination in clinic	3 (25.0)
Home insemination	1 (8.3)
In vitro fertilization	2 (16.7)
*Planning of pregnancy carried to term*	
With cis-gender female	0 (0.0)
With cis-gender male	5 (41.7)
With gender-diverse partners	2 (16.7)
As single	3 (25.0)
Unplanned	2 (16.7)
*Relationship status during peri-partum period*	
Partnered with other parent of child	8 (66.7)
Single without coparent	2 (16.7)
Single with former partner as coparent	1 (8.3)
No co-parent but in steady relationship	1 (8.3)
*Mode of delivery*	
Vaginal delivery	9 (75.0)
Elective cesarean	3 (25.0)
*Biological children*	
One child	7 (58.3)
More than one child	5 (41.7)

### Theme and Categories

The analysis resulted in one overarching theme, labeled: sustaining gender congruence during pregnancy. The three main categories: (1) considering pregnancy; (2) undergoing pregnancy and childbirth; and (3) postnatal reflections, follow a timeline for reasons of clarity. The main category, considering pregnancy, covers the period when participants were contemplating if pregnancy was a viable option for them in relation to their gender identity and the ways in which they prepared for pregnancy. It consisted of the following sub-categories: (1) negotiating pregnancy as a sign of being female; (2) identity maturation and access to gender-affirming treatment; (3) role models—mirroring, information, and identity pride. The main category, undergoing pregnancy and childbirth, covered the duration of pregnancy, childbirth, and nursing and focused on experiences of stigma, gender incongruence, and gender dysphoria. It has three sub-categories: (1) experiencing and handling the pregnant body; (2) experiencing and handling the reactions of others; (3) giving birth, and (4) nursing. The final main theme, postnatal reflections, builds on the perspectives that participants had developed as they looked at their pregnancy in retrospect. This main category consisted of the subcategories: (1) mental health problems in association with pregnancy and (2) undergoing pregnancy again. The theme and main categories are presented together with their sub-categories in Table 3.

**Table 3:** Overview of theme, main categories, and sub-categories

Theme	Sustaining gender congruence during pregnancy		
Main categories	Considering pregnancy	Undergoing pregnancy and childbirth	Postnatal reflections
Sub-Categories	Negotiating Pregnancy as a Sign of being a Woman	Experiencing and Handling the Pregnant Body	Mental Health Problems in Association with Pregnancy
	Identity Maturation and Access to Gender-Affirming Treatment	Experiencing and Handling the Reactions of Others	Undergoing Pregnancy Again
	Role Models—Mirroring, Information and Identity Pride	Giving Birth	
	Keeping an Unplanned Pregnancy	Nursing	

### Theme: Sustaining Gender Congruence During Pregnancy

The societal norm that only women get pregnant provided a backdrop as participants strived to reconcile pregnancy with their gender identity. While having a child was paramount, sustaining gender congruence was considered essential for their health and well-being. By renegotiating the gendered connotations of pregnancy, focusing on the desired outcome of becoming a parent, distancing themselves from their body and pregnancy, or trying to hide from the scrutiny of others, pregnancy became possible. The extent to which pregnancy was compatible with the gender identity of participants varied. However, existing with a pregnant body in a society where gender is assumed to be binary and congruent, and where pregnancy is normatively inseparable from femininity, forced participants to deal with gender norms regardless of whether pregnancy was in line with their gender identity or not. This was particularly evident when participants had to decide how they wanted to present their gender and pregnancy status to others.

While pregnancy resulted in a greater appreciation of the body that participants had been born with, it did not reduce the need for gender-affirming treatments. On the contrary, gender-affirming treatment was considered to make pregnancy easier as it enhanced masculinity and gender congruence during pregnancy, allowing participants to be read as male and freeing them to break gender norms, limiting their gender dysphoria.

### Considering Pregnancy

#### Negotiating Pregnancy as a Sign of Being a Woman

Having children was important, but reproductive options were limited. Consequently, all participants had deliberated if and to what extent pregnancy was possible and compatible with their gender identity, gender dysphoria and the gendered expectations of others. For almost all participants pregnancy had initially been difficult to imagine as it was associated with being a woman by participants as well as by others, as Eric said:

It is so utterly female to be pregnant in today’s society or it has always been […] the function of women […] it is what women are here for and I could not identify with that, it felt really far away.

Most participants regarded pregnancy as a pragmatic means to an end and a time to endure, since it contradicted their gender identity and violated gender norms. To cope, participants renegotiated the gendered connotations of pregnancy, reinterpreting and labeling it as masculine or a gender-neutral act. By regarding pregnancy as a gendered act that did not define their gender, insisting that it is the gender identity of each individual that determines what gender that individual has rather than their genitals or reproductive decisions, participants resisted the internalization of gender norms, making pregnancy possible as George explained:

I did want children and I thought that well, a man’s got to do what a man’s got to do […] gender role is what you do. Gender identity is who you are. I can do things that are coded as feminine. It does not affect who I am.

Normalization of pregnancy as a gender-neutral act was also achieved by using other gender-diverse individuals as reference, as Björn said: “It [pregnancy] was not a contradiction. I am a transgender man. We have existed since the beginning of time and have undergone pregnancy since then.”

While pregnancy could be successfully framed as a gender-neutral act, certain pregnancy-related body changes and the reactions of others to pregnancy as a sign of femininity were expected to trigger gender dysphoria. Three participants highlighted that they had been curious to undergo pregnancy, despite its female connotations, regarding it as an opportunity that gave their body a new value, as Charlie noted:

I have probably always wanted to – no maybe not always – but it has always been there as an alternative […] and I have been rather curious to have a living being inside your body […] I find it difficult to understand why everyone wouldn’t be curious of that, if you are born with the set of [body parts] that makes it possible.

#### Identity Maturation and Access to Gender-Affirming Treatment

All participants had grown up when sterilization was mandatory and an expected part of the gender-affirming treatment process, requiring them to prioritize between treatment and children. The desire to have children had prevented two participants from realizing that they did not identify as women, given the strong association between pregnancy and femininity, a gender stereotype that they had internalized. Eventually their gender identity and wish to have children had had to be reconciled, as John explained:

I always knew that I wanted children, and because of that it took me longer to figure out that I was transsexual. It took precedence over my gender identity. “Since I want children, how can I possibly be a man?” It was incompatible in my mind. Since having children was my greatest wish, I came to [identify as] a very masculine woman […] eventually I understood that it was not enough, I was a man. […] I read about adoption and realized that it was practically impossible. And since I have never been interested in women, which was very inconvenient, I understood that I would have to give birth. And while I had thought of [pregnancy] when I was young it was harder when I discovered my [male] identity.

This and other participants linked their readiness to undergo pregnancy to the level of maturity of their gender identity development process. Two participants had envisioned pregnancy when they still identified as women but found it difficult to do so once they had begun to question their gender identity as pregnancy opposed dominant masculinity norm that they felt obliged to live by.

Gender norm adherence was particularly important during the early stages of the gender identity realization process. Later, when participants were more secure in their identity and therefore better prepared to face the consequences of breaking gender norms, pregnancy became attainable. To access gender-affirming treatments contributed to this stability, as it increased gender congruence, making participants more comfortable in themselves and less exposed to the scrutiny and questioning of others. In a more masculine body, participants felt more prepared to challenge gender stereotypes by undergoing pregnancy. All in all this contributed to making pregnancy feasible, as Aiden recalled:

I had been on hormones a few years and had left the trauma of the gender identity assessment behind. So, life became easier, and my identity was more stable and it felt more accessible to go through pregnancy […] Once I got the idea it felt natural, or kind of possible at least. Not like a sure thing right away, but more like yes, I can do this.

The notion that gender-affirming treatments made pregnancy easier was shared by multiparous participants who had tried pregnancy without, as well as after, gender-affirming treatments and also came up in six out of eight interviews with participants who had undergone one pregnancy only. Eight participants had started testosterone before pregnancy. Two primiparous participants had done, so when they were actively contemplating pregnancy, although they were afraid that the treatment could hamper their fertility as they did not want to undergo pregnancy in a feminine body. Masculinizing chest surgery was also expected and experienced as making pregnancy easier, as mentioned by five participants. One participant had regarded access to chest surgery a prerequisite to cope with pregnancy and had therefore incorporated it into their pregnancy plans, as Iben explained:

I had planned to have a child on my own for a long time and to be able to see myself as pregnant it was necessary for me to go through a mastectomy. […] It was the start of the pregnancy, to do the operation. […] The breasts had to go […] I would never make it otherwise. Never. I would have felt so disgusted with myself, it was unthinkable.

#### Seeking Information and Identification from Role Models

Planning for pregnancy involved loneliness and a lack of information. Since knowledge and representations of pregnancies in gender-diverse individuals were missing in mainstream society and health care, finding role models to identify with and learn from was key. Participants turned to role models for information on whether testosterone would hamper fertility, child development or child health since they were unable to get this information from their healthcare provider or did not trust the response they got. Other questions included if nursing was possible after chest masculinization surgery and during testosterone treatment. John recalled how media reports about an American transgender man who had given birth after testosterone treatment, as well as conversations a friend in the transgender community, had offered hope and added to the information that his endocrinologist had provided.

When Thomas Beaty delivered […] I was stoked because I knew that he had been on testosterone for a long time and was still able to give birth to three children without any problems. I was worried about that, and during the assessment […].the endocrinologist scared me by saying that the hormones would harm the eggs […] but a friend of mine, who has contacts in the transgender community, said that he knew of several transmen who had stopped taking hormones and had had healthy children.

Role models also provided valuable insights into how pregnancy could be reconciled with their gender identity and how to deal with gender dysphoria. As Iben explained:

It was very abstract before I got pregnant, it felt unreal […] I tried to find someone like me in the literature, only to find a comic with a pregnant butch. [Upon seeing other gender-diverse people who had been pregnant] I felt such a connection to those who had walked this road ahead of me […] it felt so safe to have them.

Support from gender-diverse peers who had undergone pregnancy enabled participants to envision themselves as pregnant, prepare and make informed decisions. Identification with others decreased feelings of loneliness and insecurity and catalyzed a broadened view of masculinity, making pregnancy permissible. As participants moved forward toward the decision to undergo pregnancy, some came to identify themselves as agents of change and role models, enhancing identity pride, as Lennon explained:

I do not think of pregnancy as a gendered process […] I felt it would be a beautiful experience […] and was strengthened by the idea of a transgender person being pregnant, to make that concept possible, like I have seen others do before me, to somehow broaden the opportunities of people in building a family, it felt nice. I believe that I am influenced by the images of pregnant transgender people that have come during the last decades, that have opened those opportunities and [broadened my] ways of thinking […] It has been a process of relearning, in relation to the gender norms of society.

#### Keeping an Unplanned Pregnancy

For three participants, pregnancy was unplanned. Nevertheless, these participants decided to carry their pregnancy to term, arguing that they wanted children in the long run, reproductive options were limited, and they wished to avoid fertility preservation. Denial and a reluctance to have an abortion also contributed, as Frankie said:

I thought of having an abortion because I did not think that I could handle it [pregnancy]. But I could not stand the emotions, the guilt. So I kept her and it was a really hard time […] in the beginning I tried to laugh it off. I did not want to realize that I was pregnant […] it was only when I was three or four months pregnant that it started to dawn on me, oh I am actually pregnant […] previously I did not want any children, but I thought what if I change my mind, what if I want a child? Because I could not have a surrogate, it is not allowed. [This way] I could have a child the natural way and could avoid insemination and egg harvesting.

Pressure from others also contributed to the decision to refrain from having an abortion. David, who had always opposed pregnancy as it contradicted his gender identity, but became involuntarily pregnant after a sexual assault, recalled being talked out of having an abortion. He said:

When I started transitioning, I was asked about storing eggs [but] I had dysphoria for getting pregnant and carrying I child, because I thought that it, well, it made me feel less of a man. […] I wanted to abort when I found out that I was pregnant, but my girlfriend is religious and she was like: “You know what? Things happen for a reason. […] Maybe this is your family.” And then she was like: “Ok, if you don’t want to keep the baby, give birth! I’m going to take care of the child. Because now you are going to commit murder”. She made me feel like the devil himself. So I thought [I could] not proceed with the abortion.

A lack of trust in healthcare providers was also mentioned as a reason to refrain from abortion. A participant who had lost trust in healthcare providers after undergoing assessment when sterilization was still required to change legal gender, argued that an irrational fear that someone would forcibly sterilize him kept him from having an abortion. Two of the participants who had an unplanned pregnancy were strongly opposed to childbearing as it violated their gender identity. The fact that they decided to keep their child in spite of this, highlights that the question of whether pregnancy is compatible with the gender identity of an individual or not, is merely one of several factors that come in to play when gender-diverse individuals consider pregnancy. The interviews with these individuals show that laws and regulations that limit reproductive options such as the prohibition of surrogacy and the former sterilization requirement, exposure to sexual violence, which was the cause of pregnancy in one participant and inadequate contraception that resulted in two additional pregnancies, can be of equal or paramount importance, especially when norms and experiences discourage from abortion.

### Undergoing Pregnancy and Childbirth

#### Experiencing and Handling the Pregnant Body

Pregnancy involved dealing with a body that became increasingly feminized. The protruding belly caused little dysphoria since it was associated with the child and did not expose participants as female assigned at birth, but could pass as a beer belly or be hidden under baggy clothes. Breast development, however, was a strong trigger of dysphoria. Eleven participants, including five of the six participants who had had chest masculinization surgery, experienced breast enlargement. Being unprepared for it to happen and unable to use a binder toward the end of the pregnancy increased discomfort. Charlie, who regretted postponing chest surgery until after childbirth, envisioned pregnancy with a flat chest as less gender dysphoric, saying:

To carry a child […] is no problem in relation to my gender identity… to have a child in your belly that grows. It is everything else with the pregnancy. The female hormones but also the breasts […] they became like three times bigger. Now afterwards I feel that the plan to do the mastectomy afterwards [after childbirth] was not right. […] It had probably been much better if they had not been there.

Other causes of gender dysphoria included mood swings associated with female hormones and the cessation of testosterone, a feminine redistribution of fat tissue, vaginal bleedings after delivery and difficulties to fit into masculine clothing. Such changes were easier to accept when anticipated. To cope, participants reminded themselves that pregnancy was only temporary and a means to an end.

Four participants came to regard their body more positively during or after pregnancy as it would give them a child that they had longed for. The body that they had previously detested for being incongruent with their gender identity suddenly worked to their advantage. This gave their body a new meaning, purpose and value, as Eric recalled:

When one could see that […] there was a baby in there, it all felt right. […]. My body suddenly had a function that I had chosen […] Yes, it made all the changes OK because I knew their purpose […] and I can often feel that [this] is the point of all of this that I have been born with.

Being at peace with one’s body was associated with the ability to separate pregnancy from its feminine connotations, being able to see other gender-diverse individuals undergo pregnancy, and being read as male. By positioning pregnancy as a bodily function rather than a gendered process, physical changes, such as stretch marks, could be accepted and even cherished, as Aiden explained:

I did not think about [pregnancy] in terms of gender. It was just something that my body can do and it is amazing, so fascinating. So it was interesting and exciting to go through it. It was no problem. Perhaps also because I could see myself reflected in others, other transmasculine individuals who have also done it [been pregnant], gave me some sort of mirror in terms of masculinity. But then again, I passed as male during the entire pregnancy […] The minimal breast development that I got was the only thing that was hard to handle, but I do like the memories they are left in my body after the pregnancy. They are precious and cherished.

Other participants found the physical changes harder to deal with. They tried to forget about their body and consequently also their baby, avoiding looking at themselves or establishing a mental block to the physical sensations that reminded them of their child. This coping strategy made it difficult to connect with the baby, raising concerns regarding their later child attachment and how that would affect the well-being of their child, as Kylar explained:

It was like my body was being used for something, it wasn’t me. I distanced myself from it that way, seeing my body as a nine-month incubator to have kids, and later on it will be mine again […] but it was hard when the baby moved, to connect with the baby […] to interact with the baby through my body [I said to myself] I have to get attached to this baby, and I will, but how can I do that [if it means] reconnecting with my body?

#### Experiencing and Handling the Reactions of Others

Pregnancy made the body public goods, free to touch and comment on. Getting attention, including admiration, as pregnant and consequently assigned female at birth, was sometimes a stronger trigger of gender dysphoria than the physical changes of pregnancy. At times, others questioned the gender identity of participants because of their pregnancy, intensifying gender dysphoria, alienation and loneliness. Harley described the constant struggle to deal with other people’s gender bias, saying:

To me, pregnancy has never been something female but […] I have had to handle the reactions of others, in the health care system, transgender people, cis-people […] many have placed this norm on me and said […] that it is contradictory to get pregnant as a transgender person, like I should not want to be [pregnant]. Outside of my closest sphere, there were those who said: “Why can you not just get it that you are a woman? It does not really matter that you believe that you are something else, you are obviously [female] if you are pregnant!” […] It really hurt!

Decisions on how participants were to present their gender and pregnancy status to others involved important trade-offs. Participants often tried to be read according to their gender identity by keeping their hair short, using a binder as long as breast development admitted, and by dressing in masculine clothing. Those who had not received any gender-affirming treatments yet were often read as female, despite a masculine gender presentation, protecting them from overt gender bias but causing intense gender dysphoria and the urge to isolate. Those who had received masculinizing treatment were often perceived as men. This preserved their well-being but limited the ability to share pregnancy-related experiences with others, contributing to loneliness and a fear of exposure. When the pregnancy could no longer be hidden, these participants often stayed at home or sought sick leave to avoid exposure, stares, condemnation and fear of verbal and physical violence from strangers. Isolation toward the end of the pregnancy aimed to protect both participants and their child. Charlie, who was afraid of being kicked in the abdomen by a stranger, explained:

Being stared at when you are out in town is really not that bad, although it wears you down. But it is related to worse things. Being threatened is hard enough when you only have yourself to think about. Now you have this life inside that you must protect with your body, when you are huge and cannot run away.

While most participants hid their pregnancy, others went public to show that pregnancy is not an exclusively feminine experience. Acting as a role model offered a sense of pride and retribution, despite exposing oneself to other people’s judgment.

#### Giving Birth

Birth wishes varied. Four participants favored a vaginal delivery, regarding it as natural and healthier for their child. The others felt that a vaginal delivery did not violate their gender identity, ignored it if it did, or highlighted its masculine connotations, as George described: “I made it into a macho thing, you know like taking the bus when you go to delivery. Acting like, ‘We can do this!’ […] because giving birth is an achievement really.”

Participants who had undergone gender-affirming treatments argued that it made them feel more at home in their bodies and therefore better prepared for labor. Interpreting and labeling their genitalia according to their gender identity also helped. While we did not ask participants what words they used to refer to their genitalia, the positioning of their genitalia as masculine came up in interviews, as Harley said:

The body parts, inner and outer, I do not think of them as feminine. I do not have female body parts, because if I have them as a transgender man, then they are male body parts […] I do not become less of a transgender man for […] giving birth vaginally.

Reasons to prefer a vaginal birth also included a lack of trust in healthcare professionals, which made participants reluctant to give up control and be anesthetized during a cesarean. Two participants aimed to avoid a cesarean section since its characteristic scar could expose them as female assigned at birth later in life. While having scars from chest masculinization surgery could also expose them as transgender, that procedure was regarded as inevitable as it reduced signs of femininity and was central to their gender congruence, while the scar from a cesarean could be avoided and marked them as female. Those who favored a cesarean envisioned a vaginal delivery as traumatic since it would expose their body in the delivery room and require them to reconnect with it during labor, triggering gender dysphoria. As Charlie explained:

Finally I just [said], I cannot give birth to this baby. You have to take it out. And it was really only about gender dysphoria […] I cannot be with my body that way […] those mega breasts and everything, I was kind of shut off, the relationship between mind and body […] if the delivery doesn’t go well, but also if it does, it could become a bloody trauma really.

Those who had distanced themselves mentally from their body feared that they would be unable to collaborate with their midwife during labor, impeding medical safety. Eric linked his derealization during a previous vaginal delivery to gender dysphoria, saying:

I was not able to tell them what I needed and I did not understand [what was happening]. I just thought that I would die […] I had no contact with those ten people standing around me […] I think it was [because gender] dysphoria also involves not identifying with your biological sex […] I did not understand that I was giving birth […] I think I got that dysphoric because it is my genitals we are talking about, and that made the brain shut off.

Most participants were happy with their delivery, giving birth the way they wanted to. Those who desired a vaginal delivery found their gender dysphoria manageable, while those who had a cesarean felt relieved. One participant who was denied an elective cesarean had a vaginal delivery marked by dissociation, pain, feelings of helplessness and pronounced gender dysphoria.

#### Nursing

Baby-feeding preferences varied. Half of the participants had tried to nurse, while the other half did not. Those who chose bottle feeding, said that it was equivalent to nursing and supported their gender congruence. Those who opted to nurse argued that it was considered natural and therefore best for their baby. Two participants were surprised that nursing did not trigger any, or only very mild, breast dysphoria. Others had to distance themselves from their body and baby to nurse, raising concerns regarding child attachment. Kylar explained:

I wanted to feel properly for this baby, but to nurse at the same time was really hard […]. It is difficult to relate to another person while simultaneously shutting off the body […] I have to go on autopilot, to disconnect the body and not feel so much. I will remain close to the baby and do everything that I should, but I cannot feel too much for it, because then I will not be able to continue nursing […] it became a strategy to keep up nursing […] When I nurse it isn´t my body. It makes [me] distant to the child.

Participants who had undergone chest masculinization surgery were surprised to find that they started lactating. Five participants felt guilty about refraining from, or wanting to give up, nursing and were relieved when milk production ceased. Others wanted to nurse and wondered if they would be able to. Medical staff were unable to provide guidance of how chest masculinization surgery would affect their nursing capacity. Those who tried to nurse were able to do so. Although milk production was limited after surgery, none who had undergone mastectomy expressed regret about the procedure.

### Postnatal Reflections

#### Mental Health Problems During Pregnancy and the Peri-partum Period

While this study did not systematically investigate mental health problems, ten participants spontaneously reported anxiety and symptoms of depression, mood swings and relapse of former mental health issues such as PTSD during pregnancy. Seven participants mentioned having been diagnosed or treated for mild to severe depression during pregnancy or shortly after giving birth. Two participants described psychotic symptoms post-partum. Suicide ideation was experienced by three individuals, but no suicide attempts were conveyed.

Participants attributed their mental health problems to the stress associated with breaking gender norms and having their gender identity and right to become a parent questioned by others, anticipating discrimination, hurtful comments and fear of violence. There was also a feeling of increased sensitivity to such events during pregnancy which made it worse. Those who had experienced previous negative healthcare encounters because of their gender identity and desire to undergo pregnancy were hypervigilant of ill treatment in association with healthcare visits, which strained their mental health even further. In the end it all added up, culminating in depression or anxiety. Björn, who experienced that the gender team had tried to persuade him to undergo sterilization during his evaluation for gender dysphoria and gender-affirming treatment, which took place at a time when sterilization was still legally required to change legal gender in Sweden, explained how this experience made it stressful to seek care. He said:

I was so traumatized by my experience [with the gender team that] I did not even attend primary health care for several years. [But I had to] go to regular check-ups for the sake of the baby. […] I developed trust in one of the midwives but as delivery approached and you have to go to the hospital and, well, that was really scary. […] I have post-traumatic stress after the experiences with the gender team and it gets worse when you are pregnant because you get more sensitive. All those memories and fears come back. […] I had post-partum depression. It was because of the 15-year battle to have a child, uncertainty, and fear, and then suddenly it works out. By then I was so exhausted […]. They put me on antidepressants, and it helped.

Hormonal effects of pregnancy and the cessation of testosterone also strained the mental health of participants. Charlie recalled the mood-altering effects of endogenous hormonal production during pregnancy, saying:

It was really hard with the hormones, getting so emotional, almost like a caricature of femininity. Without testosterone I have a sensitivity to hormones, like PMS [Premenstrual syndrome]. The biggest problem was becoming emotionally unstable and losing control. [After giving birth] I was in a really, really bad shape mentally, because of the hormonal shift.

Pausing testosterone made participants more susceptible to the judgment of others, both because its masculinizing effect wore off, but also because their feelings became attenuated. This made it harder to shield oneself from other people’s opinions, a vulnerability that coincided with signs of depression, as Aiden explained:

[Going off testosterone] affected me mentally. I was not myself during the time I was pregnant and felt depressed, ate less, reacted to things in a different way […] all my feelings came closer, it was as if my skin was thinner, making it harder to protect myself from what happened around me.

While gender norms and the reprisals associated with breaking them were regarded as a cause of mental health problems, participants also described paying closer attention to, and becoming more susceptible to, gender norms during depressive episodes, lowering their mood even further and exacerbating their gender dysphoria. Gender identity-related self-stigma and gender dysphoria were experienced as more intense during depressive episodes. As Frankie, who was offered antidepressants, said:

I used to lay in the bathtub to keep calm, always focusing on something, so that I did not have to think about my body. […] I tried to [keep myself busy] otherwise you get stuck with your thoughts and just break down, pondering why was I born this way, why can’t I just be normal […] because one does not want to be transgender. You want to be born like a regular human being, feel normal.

During episodes of depression and anxiety, participants questioned their ability protect themselves and their child against other people’s gender bias and blamed themselves for having a child that might be ostracized. Depression combined with increased difficulties to protect oneself from interpersonal and self-stigma also affected the ability of participants to claim their gender identity in relation to others. One participant did not disclose to others that he had given birth to his child as he was afraid that this would make others regard him as less of a man, inventing a story of how he became a father. Two other participants recalled how they had begun to question their right to label themselves fathers after being diagnosed with post-partum depression, as John said:

It was a total, total collapse, which they labelled severe post-partum depression and identity crisis[…] I did not feel like a mother, definitely not, did not feel like a dad – or I did feel like a dad but I did not dare to, I somehow felt as if I did not have the right to call myself dad, there was this uncertainty […] do I have the right to label myself a man just because I am?

After being admitted to psychiatry and receiving electroconvulsive therapy, this participant was able to reclaim his male gender identity, highlighting how depression increased his vulnerability to gender norms and trans negativity. While several participants received meaningful psychiatric care, others struggled to find providers with sufficient knowledge of gender dysphoria to support their mental health.

#### Undergoing Pregnancy Again

Participants summarized their experiences of pregnancy by asking themselves if they would be willing go through it again. Among the eight primiparous participants, four were reluctant to undergo another pregnancy, wanting to avoid mental health problems and recurrence of gender dysphoria. Those who considered a second child expected their next pregnancy to be easier since they knew what to expect and had been able to integrate pregnancy into their sense of masculinity. Further gender-affirming treatment was also expected to help, as Frankie explained:

While it is impossible to take testosterone during pregnancy, it is possible to take it before [my next pregnancy]. I was telling my partner that if I grow a beard, I will have no problem undergoing [another] pregnancy. I don’t think so, definitely not. Because then one might read it as a beer belly, or that I am a little fat. Then [I] would not have to constantly think about my body.

The five participants of this study who had been pregnant more than once and had given birth or were about to give birth, all experienced their later pregnancies as easier to deal with. Coming out to themselves and others as transgender, meeting other pregnant transgender men, adopting a masculine gender expression, being read according to their gender identity, feeling able to withstand the scrutiny of others and knowing what body changes to expect decreased gender dysphoria and made pregnancy less challenging. Eric explained:

I found it easier [to be pregnant] with my second child since I had come out and had come to understand [my gender identity] myself […] I dressed more masculine […] and knew what to expect [like] now my body will do this.

Gender-affirming treatments had also made pregnancy an increasingly positive experience, as George recalled:

[In my first pregnancy] my breasts were swollen, and I had not received the [gender-affirming treatment] that I really needed. So, I still had my [feminine] voice and everything, I had not been able to start any hormonal treatments yet […] The second time was easier perhaps because I was used to it and also because I knew that I would have access to treatment afterwards […] This [third time] it really felt great because I had no breasts.

## Discussion

This qualitative retrospective interview study aimed to investigate how gender-diverse individuals in Sweden experience and handle pregnancy, childbirth and nursing in relation to their gender identity to gain a deeper insight of how pregnancy can be reconciled with a masculine or non-binary gender identity and to identify factors that can be used to support a healthy pregnancy. We found that the norm that you have to be a woman to bear a child, combined with the risk of increased gender dysphoria, deterred from gestational pregnancy and strained the mental health of participants. However, factors that promoted gender congruence during pregnancy, such as mirroring and information from role models, access to gender-affirming treatment and the ability to renegotiate childbearing as a gendered process, could make pregnancy a tolerable or even rewarding experience.

Early in their coming-out process, participants found it difficult to imagine themselves as pregnant. This can be explained by their internalization of dominant gender norms that treat pregnancy in men as something unintelligible (Hoffkling et al., 2017) as pregnancy is strongly associated with cis-gender women in western society (Besse et al., 2020; Charlton et al., 2021; Charter et al., 2018; Lampe et al., 2019). As individuals seek to present their gender based on social expectations (Lampe et al., 2019; Westbrook & Schilt, 2013), and pregnancy in gender-diverse individuals opposes the normative assumption of sex and gender congruence (Wenzlaff et al., 2018), undergoing pregnancy appeared to be too challenging and could expose participants to interpersonal stigma and questioning of their gender identity. In the context of the present study, it is also likely that the Swedish legislation, which required sterility for anyone who wanted to change their legal gender until its amendment in 2013 (Dhejne et al., 2014), affected what reproductive goals participants could visualize during the beginning of their transition. Later on, when they had grown to accept their gender identity, had received gender-affirming treatment and were less exposed to and affected by the opinions of others, pregnancy was easier to envision and cope with. Previous research on experiences of pregnancy in gender-diverse individuals (Besse et al., 2020; Chen et al., 2018; Ellis et al., 2015; Falck et al., 2021; Hoffkling et al., 2017; Light et al., 2014; van Amesfoort et al., 2023) has not analyzed the ability to imagine oneself as pregnant in the context of identity maturation, although an Australian study found that transgender men who felt pressured into motherhood when growing up were able to see themselves as potential fathers upon disclosure of their gender identity (Charter et al., 2018). Descriptions of the transgender coming-out process indicate that it is a long-term journey in which mirroring and acceptance from others are essential in the initial phases, while tolerance for ambiguity, self-acceptance and confidence characterize the latter integration stage (Bockting & Coleman, 2016). The finding that reproductive aims may change throughout the coming-out process indicates that patients undergoing evaluation for gender dysphoria might not be able to foresee what reproductive aims they will have, and be able to cope with, later in their transition. This may be a central message to convey when counseling gender-diverse individuals on fertility preservation and parenthood as part of the evaluation for gender-affirming treatment.

In the context of treatment assessment, it is also interesting to note that testosterone and chest surgery made pregnancy and vaginal delivery easier to envision and cope with, as they enhanced gender congruence during pregnancy, according to the results of this study. This finding adds to existing research on the treatment of gender dysphoria in presumably non-pregnant female-assigned individuals, linking improvements in body satisfaction and mental health to the use of testosterone (Fisher et al., 2016) as well as chest masculinization surgery (Owen-Smith et al., 2018). It also reinforces a previous finding that chest masculinization surgery is regarded a prerequisite for pregnancy by some individuals (MacDonald et al., 2016), and that testosterone treatment before conception can validate a masculine gender identity in individuals who later chose to undergo pregnancy (Charter et al., 2018). The present study indicates that the increased gender congruence that gender-affirming treatments result in before pregnancy can be carried forward into the pregnancy. While gender-affirming treatment was inevitable to undergo pregnancy for some participants, others had postponed such desired treatment due to fear that testosterone could affect their fertility and child health negatively. As the shortage of information obstructed reproductive decision-making, participants called for improved information, in line with previous studies (Hoffling et al., 2017; van Amesfoort et al., 2023). While the precise effects of testosterone on fertility remain to be decided (McCracken et al., 2022; Rodriguez-Wallberg et al., 2022), clinicians should familiarize themselves with the best available knowledge and follow existing guidelines on reproductive counseling before the initiation of testosterone treatment (Coleman et al., 2022; Hembree et al., 2017), carefully weighing the risks, benefits and ideal timing of gender-affirming treatment for each individual patient.

The lack of information on how gender-affirming care could affect reproduction and what pregnancy might be like, combined with the invisibility of gender-diverse gestational individuals in mainstream society, made the participants of this study feel odd and alone. To avoid interpersonal stigma and gender dysphoria, they isolated themselves, enhancing their loneliness even further. The finding that pregnancy in gender-diverse individuals is characterized by loneliness and feelings of otherness due to a shortage of information and representation is supported by previous studies (Besse et al., 2020; Charter et al., 2018; Ellis et al., 2015; Hoffkling et al., 2017; Lampe et al., 2019; Light et al., 2014; van Amesfoort et al., 2023). In contrast, in the cis-gender population mothers report lower levels of loneliness in the form of social inadequacy, alienation and interpersonal isolation during pregnancy and early motherhood, as compared to women in the general population (Rokach, 2004), as well as a lower risk of loneliness due to mistrust, low self-esteem and social marginalization compared to women without children (Rokach, 2007). As loneliness has been found to predict post-natal depression (Luoma et al., 2019) as well as chronic depression in mothers (Luoma et al., 2015), it is central to prevent.

To combat feelings of alienation, loneliness and a lack of information, participants stressed the importance of role models. By establishing contact with role models, they could envision themselves as pregnant, gain knowledge and prepare for pregnancy-related changes. Being a role model to others also enhanced identity pride and made pregnancy a more positive experience. Research shows that peer support can improve self-esteem and social participation, help individuals gain control of their life and decrease anxiety (MacLellan et al., 2015). By using one’s own experiences to help others, painful memories gain meaning and can be integrated into a sense of self, increasing self-acceptance (MacLellan et al., 2015). While peer support to reduce loneliness in mothers shows mixed results (Nowland et al., 2021), other qualitative studies on pregnancy in gender-diverse individuals have also suggested that role models may normalize trans pregnancies and combat isolation (Hoffkling et al., 2017; Lampe et al., 2019; van Amesfoort et al., 2023). As knowledge and positive portrayals of pregnancy in transgender men and non-binary individuals remain scarce in health care and general society (Falck et al., 2021; McCracken et al., 2022; Obedin-Maliver & Makadon, 2016), prenatal health services and gender teams should consider involving peer supporters with a history of gestational pregnancy in their programs and study the effectiveness of such initiatives in reducing gender incongruence and loneliness during pregnancy.

Participants experienced increased gender dysphoria as pregnancy progressed. Breast enlargement was a particularly strong trigger of dysphoria, in agreement with previous research (Charter et al., 2018; MacDonald et al., 2016; van Amesfoort et al., 2023), as can be explained by the strong association between breasts and femininity (Charter et al., 2018; Spencer, 1996). Breast growth occurred also after chest masculinization surgery, which participants were unprepared for. As physical changes that could be foreseen were easier to handle, provider-led information on how the body transforms during pregnancy, including after gender-affirming treatment, was requested. Some participants who used their breasts/chest to feed their baby were surprised to find that nursing did not trigger their breast dysphoria, confirming results from earlier studies (MacDonald et al., 2016; van Amesfoort et al., 2023). Those who wished to nurse after chest masculinization surgery were able to do so, although lactation was limited. While the effects of a mastectomy may include reduced milk production, a smaller nipple and tighter tissue which the baby might not be able to latch on to (MacDonald, 2019), no participant expressed regret over their chest masculinization surgery. To our knowledge, existing studies on experiences of pregnancy in gender-diverse individuals have not examined the issue of treatment in relation to child-feeding practices, although a Dutch study noted that a participant who had undergone chest masculinization surgery at an early age wished that he had understood that this would prevent him from nursing a child later on (van Amesfoort et al., 2023). Since the timing and type of surgical technique are likely to affect nursing capacity, risk of breast enlargement, and mastitis, patients requiring chest masculinization surgery should be informed of these aspects prior to the procedure. As patients may fear that access to surgery can be limited if they convey an interest in nursing (Falck et al., 2021; MacDonald et al., 2016), and chest masculinization surgery may be a necessity to cope with pregnancy for some individuals, which this and a previous study show (MacDonald et al., 2016), healthcare providers should take charge in raising the subject.

Some participants handled their increased gender dysphoria from pregnancy and nursing by establishing a mental block to their body and baby. The strategy to detach from one’s body also involved efforts to block out the fetus as it moved in the uterus, or the infant when it was nursing, highlighting how the body discomfort that participants experienced was extended to their child, raising concerns on parent–child attachment and child health. To detach from the body to cope with pregnancy has been described in transgender men in a couple of previous studies (Charter et al., 2018; Ellis et al., 2015) and may include a lack of connection to the developing fetus (Ellis et al., 2015). However, to our knowledge it has never been described during nursing. While body detachment can be an adaptive coping strategy to handle pressing stressors (Elklit, 1996; Roger et al., 1993), its effects on child health are yet to be decided. Parent–child attachment builds on the ability of the caregiver to respond to the child’s signals (Newman et al., 2015) and is likely to be negatively affected if parents frequently distance themselves from their own body and child due to gender dysphoria. Since parent–child attachment is essential for child development (Erickson et al., 2019), gestational gender-diverse individuals may need assistance to connect with their body and baby after delivery. The finding that individuals may resort to detachment to force themselves to nurse their child, believing that their own milk is superior for the health of their baby, calls for nuanced information and individualized counseling on baby-feeding choices.

Existing literature on reproduction and obstetrics does not often include experiences of childbirth in gender-diverse individuals, and very few studies have had this focus (Besse et al., 2020). In line with a previous study (Ellis et al., 2015), participants expressed varying birth wishes. Some associated a vaginal delivery with an increased risk of gender dysphoria and dissociation and wanted an elective cesarean to avoid this, while others preferred a natural birth, regarding this as beneficial for the health of their child. An interesting and potentially novel finding was the reluctance to undergo a cesarean in order to avoid scarring that would expose participants as assigned female at birth. Fear of mistreatment by healthcare providers in association with childbirth, which has been described in gender-diverse individuals in previous studies (Falck et al., 2021; Malmquist et al., 2019), was a reason to prefer a cesarean as well as a vaginal delivery. Studies on pregnant women show that approximately 6–15% have a severe fear of childbirth (Nilsson et al., 2018). Gender-diverse individuals share the same fears of childbirth as women, but have additional reasons for anxiety such as fear of mistreatment or exacerbation of gender dysphoria during childbirth (Besse et al., 2020). Research on expectant mothers shows that fear of giving birth is a risk factor for post-traumatic stress symptoms one month after delivery (Ayers et al., 2016), may affect uterine blood flow (Aksoy et al., 2016) and is associated with increased levels of serum cortisol (Alder et al., 2011) which may affect child development negatively (Beijers et al., 2014). Reducing stress is thus essential both for the health of the gestational parent and for their child. Moreso, the risk of a traumatic birth increases if the individual giving birth is afraid or feels threatened (Slade, 2006), was exposed to previous trauma, has mental health problems or experiences limited choice or loss of control during birth (Greenfield et al., 2016: O´Donovan et al., 2014). As all these factors are more likely to occur in gender-diverse individuals (Greenfield & Darwin, 2021), it is important to be attentive to their wishes, needs and experiences when selecting the appropriate method of birth. As the risks of a cesarean and a vaginal delivery are weighed,fear of giving birth due to gender dysphoria or a lack of trust in healthcare providers is important to consider. As participants reinterpreted pregnancy, delivery and their genitalia as masculine or non-binary according to their gender identity, clinicians may also consider asking their patients what words they use to refer to gendered body parts to help reduce gender dysphoria during delivery. This may involve using words such as penis when referring to body parts that are normatively positioned as female (Zimman, 2019), or adopting new concepts invented by the patient or the transgender community such as “mangina” (Ragosta et al., 2021) to aid inclusion.

Although our study protocol did not specifically target psychiatric problems related to pregnancy, this theme surfaced spontaneously and repeatedly in participant narratives. Participants associated their mental health conditions such as perinatal depression and anxiety with the stress that came from breaking gender norms, anticipating and experiencing stigma in daily life including in healthcare encounters. This finding supports the minority stress model, which holds that stigma acts as a stressor that drives morbidity and mortality in marginalized populations (Meyer, 1995; Nadal et al., 2014) and elevates the risk of depression and anxiety among transgender individuals (White Hughto et al., 2015). Participants reported an increased susceptibility to gender norms and interpersonal stigma during depressive episodes, which lowered their mood and exacerbated their dysphoria. Previous research has found that depression potentiates the processing of negative feedback about oneself (Beck, 1974; Mueller et al., 2015), which may explain why participants experienced increased gender dysphoria and self-stigma during periods of depression. Depression is associated with a reduced cognitive and emotional function (Richards, 2011) as well as an impaired ability to imagine the future coupled with a low belief in the ability to reach desired goals (Hallford, 2019; McGrath & Repetti, 2002; Muris, 2002; Roepke & Seligman, 2016; Yang et al., 2022). This may explain why participants doubted their ability to protect their children from the bias of others during depression as well as why they found it harder to claim authority over their gender identity and as fathers when suffering from post-partum depression. The inability to stand up for one’s gender identity in relation to others during depression is a novel and potentially important finding, highlighting the need to further assess how affective disorders may interact with symptoms of gender dysphoria.

Aside from psychosocial risk factors, participants found the cessation of testosterone problematic for their mental health, describing mood swings as well as increased gender dysphoria resulting from feminization and increased psychological sensitivity. Such withdrawal symptoms have been associated with the termination of testosterone before pregnancy in previous studies (Charter et al., 2018; Davis & St Amand, 2014). Since testosterone treatment in gender-diverse individuals is associated with a lowered risk of depressive symptoms (Fisher et al., 2016; Owen-Smith et al., 2018; St. Armand et al., 2011), it is possible that pausing such treatment could increase the risk of depression in gestational gender-diverse individuals who are attempting to conceive. This is another finding that deserves to be followed up in future studies.

All in all, a minimal number of studies have discussed perinatal mental health concerns in gender-diverse individuals (Charter et al., 2018; Greenfield & Darwin, 2021; Hoffkling et al., 2017; Light et al., 2014; Malmquist et al., 2019; Stroumsa et al., 2019; van Amesfoort et al., 2023). While it remains to be decided if gender-diverse individuals are at a higher risk of perinatal depression and other perinatal mental health problems, this appears likely (Greenfield & Darwin, 2021; Kirubarajan et al., 2022). A recent study reported a higher prevalence of depression and anxiety among gestational transgender men and individuals diagnosed with gender dysphoria who had given birth between 2014 and 2018 compared to mothers who had delivered during the same time period (Stroumsa et al., 2023). Unfortunately the authors did not provide information as to whether depression or anxiety were preexisting conditions or occurred during the peri-partum period. Perinatal depression has prevalence rate of up to 20% in women (O'Hara & Wisner, 2014). Risk factors include a previous episode of depression, anxiety or bipolar disorder (O'Hara & Wisner, 2014), all of which have a higher prevalence among gender-diverse individuals than the general population (Socialstyrelsen, 2020). Perinatal depression has also been linked to stressful life events, poor self-esteem and a lack of social support (O'Hara & Wisner, 2014), which are more likely to occur among gender-diverse individuals (Greenfield & Darwin, 2021). Furthermore loneliness, which was a prominent feature of pregnancy in this as well as in previous studies (Charter et al., 2018; de Castro-Peeraza et al., 2019; Ellis et al., 2015; Hoffkling et al., 2017; Obedin-Maliver & Makadon, 2016; Light et al., 2014; van Amesfoort et al., 2023), has been associated with perinatal depression in women (Bonari et al., 2004; Leung & Kaplan, 2009). As the risk of mental health problems in gender-diverse individuals may increase during pregnancy and childbirth (Greenfield & Darwin, 2021), screening for mental health concerns such as post-partum depression is warranted. Since gender-diverse individuals report a low trust in healthcare providers (Falck et al., 2021; Kearns et al., 2021; Linander et al., 2019), which may limit their health-seeking behavior (Cruz, 2014; Zeluf et al., 2016), healthcare providers need to take a proactive role in assessing and supporting their mental health during the peri-partum period. Whereas many gender-diverse individuals have no mental health problems (Socialstyrelsen, 2020), those who do have a history of psychiatric conditions should be closely monitored for recurrence.

On a final note, while the question of how an individual can choose pregnancy and still identify as a man or non-binary was central for all participants in this study, it was only one of several factors that affected their decision to undergo pregnancy. Participants who kept an unplanned pregnancy to avoid abortion, fertility preservation and regret, and who conceived against their will, indicate that a variety of factors affect the reproductive decisions that gender-diverse individuals make. For many, pregnancy may be the only option to become a parent. This warrants clinicians and researchers moving beyond the question of whether pregnancy can be compatible with a masculine gender identity or not, and focusing their efforts on factors that can make pregnancy a tolerable or even enjoyable experience in gender-diverse individuals. It calls for broad and inclusive reproductive health services that enable gender-diverse individuals to decide if, when and how they want to have children, enhanced support systems during pregnancy and early parenthood, as well as laws that limit stigma and discrimination against this patient group. Providing guidance and understanding of how pregnancy can be renegotiated to fit a masculine or non-binary gender identity may also be helpful.

### Limitations

This study covered a broad range of participants with varying identities, age, ethnicity, relationship status, mental health conditions and functionality living across Sweden. The inclusion of multiparous participants who had experienced pregnancy during different stages of their social and medical transition was an important asset, as well as the participation of individuals who had experienced an unwanted pregnancy. An important limitation is that the study only included individuals who had carried a pregnancy to term, excluding those who did not have any children due to abortion or miscarriage. This may have resulted in an overly optimistic picture of what pregnancy as gender diverse may be like. Furthermore, none of the participants had planned their pregnancy with a cis-gendered female partner. This limits the analysis of how gender norms affect the decision to undergo pregnancy. However, the fact that we could not identify any gender-diverse individual who had undergone pregnancy while having a female partner may also be an indication of the norm that women bear children. When we recruited participants to this study, a couple of individuals approached us to say that although they wanted to participate in the study, they could not do so due to traumatic memories of giving birth. It is likely that their experiences would have added additional information concerning mental health challenges, the occurrence and management of obstetric complications, as well as gender norms in obstetric care. A more detailed account of how participants experienced and handled the interactions with healthcare providers, including of how distrust of healthcare providers may extend to maternal and obstetrical care, has been discussed in a separate article (Falck et al., 2021) and is therefore not in focus here.

In retrospect, this study would also have gained from a more systematic exploration of psychiatric diagnoses among the participants. Since perinatal psychiatry in transgender men and non-binary individuals remains thoroughly understudied and surfaced repeatedly in participant narratives, we have chosen to include results on mental health problems despite the fact that we lack comprehensive information on psychiatric diagnoses prior to pregnancy among participants.

### Recommendations for Future Research

The literature on pregnancy in gender-diverse individuals is growing but remains scarce. More knowledge on the pregnancy and birth rate among gender-diverse individuals is needed, as well as a deeper insight into the potential effect of testosterone treatment on pregnancy and reproductive outcomes, including on potential long-term effects on the offspring. The prevalence and risk factors for post-partum depression and other perinatal mental health problems, such as traumatic birth experiences in gender-diverse individuals, warrant further research. It would also be interesting to see how testosterone use affects mental health problems post-partum, as well as whether peer support can combat loneliness and contribute to healthy pregnancies in transgender individuals. The ability to assert one’s gender identity during depression, including post-partum, should be examined further. Finally, this and previous studies on gestational pregnancy in gender-diverse individuals leave no or very limited information on potential differences between binary and non-binary individuals. With the exception of a recent study (Fischer, 2021), studies focusing on how non-binary individuals experience pregnancy and child birth are missing and should be explored in additional studies.

### Conclusions

Whereas some gestational transgender men and non-binary individuals find pregnancy, childbirth and nursing fulfilling and in line with their gender identity, others suffer substantial gender dysphoria and mental health problems due to physical changes during pregnancy, the cessation of testosterone, as well as gender stereotypes that associate pregnancy with femininity. Some of these health adversities may be prevented by initiatives to normalize pregnancy in gender-diverse individuals, as well as efforts to strengthen gender congruence prior to or during pregnancy. Such attempts may include the use of role models as well as access to gender-affirming treatment and information on what pregnancy as transgender may be like. These results call for a timely treatment of gender dysphoria as well as comprehensive reproductive healthcare services that are adapted to the needs and realities of gender-diverse individuals. Laws and policies that limit stigma, discrimination and violence toward gender-diverse individuals and allow them a variety of ways to reach parenthood are also warranted. Furthermore, the risk and management of perinatal mental health problems among gestational transgender men and non-binary individuals require further attention in research and clinical practice.

## Data Availability

The data and code underlying this article cannot be shared publicly to protect the privacy of the individuals that participated in the study. The data will be shared by reasonable request to the corresponding author.
